# What Has Been Done by the Profession in France on Pyorrhœa Alveolaris

**Published:** 1895-11

**Authors:** C. N. Peirce

**Affiliations:** Philadelphia


### WHAT HAS BEEN DONE BY THE PROFESSION IN FRANCE ON PYORRHŒA ALVEOLARIS.[1]

BY C. N. PEIRCE, D.D.S., PHILADELPHIA.

It is now something over two years since considerable interest was manifested on the subject of pyorrhœa. Many papers have since been written, and much notice has been given the subject in the journals, but to-night I propose to present the views of a few of the early writers and some more recent ones of France, that these may be contrasted with what has been already published.

That pyorrhœa alveolaris is not a disease of recent origin or due to modern constitutional states alone is very evident from examinations of the skulls of ancient races. The alveolar process of crania, widely separated in time and locality, exhibited marked impairment in structure and hold a very close resemblance to that presented by more modern processes which were known to have been the seat of pyorrhœa during life. The earliest dental surgeon to record his observations was M. A. Fauchard, who, as early as 1746, fully recognized the disease in all its essential features, but failed to designate it by any specific name or term.

M. Jourdain, in 1778, described the disease as "a conjoint suppuration of the gums and alveoli." Regarding the inflammation and suppuration as expressions merely of a constitutional scorbutic state, he advised "the extraction of the teeth as affording the only possible means of cure."

M. Joirac, in 1823, called the attention of the surgical world to this form of inflammation, which, from its most characteristic symptom, the flow of pus, he gave the name of *pyorrhœa interalveolo-dentaire*. That he recognized this disease seems evident from the statement that the gums became swollen and spongy, the suppuration abundant, the teeth loose and painful, and finally fall from their sockets.

---

[1] Read before the Odontological Society of Pennsylvania, September 14, 1895.

M. Marchal de Calvi, in 1860, spoke and wrote of the disease as a gingivitis, having for its results the expulsion of the teeth, —"gingivite expulsive" he termed it. He regarded the disease as hereditary and caused by the deposition of tartar, also that it was incurable unless all the teeth were removed.

In 1867, Dr. E. Magitot published his paper, which was the most complete systematic description of the symptoms and pathology of the disease which had up to this time been found in print. He commences by stating "that under certain circumstances individuals experience a premature, progressive, and continued loosening of one or more teeth, accompanied with abundant suppuration of the alveoli, with inflammatory phenomena of the gums, fungus excrescences, abscesses, and other peculiar phenomena, without the teeth themselves presenting any apparent structural change, and that this affection, if left to itself, finally ends in the loss of these organs." Magitot is fully impressed, as a result of his studies, that the gum, being in all cases only attacked subsequently, is not the real seat of the lesion. The disease with which we are occupied, he says, "seems essentially characterized, from an anatomical point of view, by a slow and progressive destruction of the periosteal membrane,—a destruction of an inflammatory character, of chronic progress, proceeding from the neck to the end of the root, and leading, without fail, to the loss of the tooth. This special feature, its mode of origin, and the precise seat of the lesion, seem to justify the name *alveolo-dental periostitis.* But notwithstanding its primary origin in the periosteum and its complications with the gum and bony alveolar wall itself, the study of the successive morbid phenomena does not allow us to admit, as various authors have claimed, *that these parts are originally the seat of the disease.*

We should only need for proof the isolated localization of the trouble in one or more teeth without necessarily being communicated to those adjoining, the situation of the anatomical lesion, the special sign of the lesion, and, lastly, of the fact of constant cure following the removal of the affected tooth. These reasons, he says, "should more than sufficiently prove that it is the tooth and not the gingival tissue or any other part which should be regarded as the seat of the disease." Dr. Magitot then gives a clear description of the disease's progress, which is not necessary to mention here, beyond his etiology, which he gives as follows:

*Etiology.*—"The causes of this affection are quite complex, and should often be looked for, not in a local condition of the mouth or gums, but in certain conditions of the general health.

"The disease ordinarily attacks either one of the teeth singly, or several of them; but in this latter case the teeth affected are not necessarily contiguous.

"The age at which this affection appears is generally neither adolescence nor advanced age. The usual period is from thirty to fifty years. It seems equally frequent in men and women, and in the latter it often appears among the complex phenomena of the menopause.

"Certain intestinal phenomena are observed either in coincidence or in etiological connection; habitual constipation is noticed in affected patients. A physician of the Paris hospitals, Dr. Vidal, has noticed that these same patients often show dyspeptic phenomena. Perhaps these were due to difficulties of mastication. In all cases we have had personal opportunity of verifying this assertion. Gouty and rheumatic persons often show it. Those attacked by anæmia as a result of long illness are in the same condition, but there are no general troubles which exercise a more serious influence in producing the disease than albuminuria, and especially diabetes. By the first we mean here, of course, not symptomatic albuminuria, but Bright's disease, properly so called.

"In glycosuria this phenomena is absolutely continuous, and even constitutes one of the earliest signs of the diseased condition. We find, in fact, in the description of most authors, that at the beginning of diabetes the teeth are observed to loosen and decay. This assertion, as regards caries, is not correct, but the first is perfectly so, and corresponds to the osteo-periostitis, which follows in its development the same progress and advance as the general disease to reach the terminating period of the latter in the loss of a considerable number or all of the teeth."

The treatment of pyorrhœa, as suggested by Dr. Aguilhon de Serran, is especially interesting. He speaks as follows:

"I shall limit myself to a short discussion of the nature of the lesions and the treatment by means of which I have succeeded in conquering them.

"Suggestions for treatment are naturally derived from anatomo-pathological study. In the first place you must relieve the fibrous mass of the liquids which separate it, then excite the vasomotor action and favor the formation of new vessels and fibrous tissues. Chromic acid fulfils these indications to a certain point: it hardens and retracts the fibrous tissue and acts on the membrane as a powerful revulsive, and has therefore, in the hands of Dr. Magitot, given very good results. But it only acts superficially, and is only of use at the beginning of the disease. Besides, it cannot destroy the purulent burrows, the principal cause of the continuance of the trouble.

"The other methods of treatment have fallen into disuse. In most cases it is necessary to extract the teeth, one after another; and, as they are generally free from all decay, patients only decide to have it done after suffering for years, or when the stomach becomes affected as a result of insufficient mastication.

"After many fruitless attempts I had recourse to a very simple mode of treatment, which answers at once the several indications of which I have spoken. It consists in traversing the bottom of the burrow by a seton of floss silk which is left in place. It causes no inflammatory trouble. From the first the patient experiences great comfort. The unstable teeth, projecting outward, are rapidly brought back by the contraction of the fibrous tissue. Their firmness is perceptibly increased from the first hour.

"I have treated eleven patients in this manner, and I have always obtained the cessation of all pain and the strengthening of the teeth, in proportion, of course, to the extent of the destruction of the socket.

"As regards suppuration, the results are less satisfactory. It was only entirely stopped in two patients,—those in whom the disease was furthest advanced.

"I was anxious to present to you a lady whose case is curious from several points of view. Unfortunately, she is prevented by rheumatism from being present. When she consulted me this lady had been suffering for ten years. She could only eat soaked bread. She has only three molars, but she has all her incisors and three canines. She was treated for a long time with chromic acid. She even applied it so often that the roots, almost entirely exposed, are dyed a deep green. A few setons were sufficient to cause the disappearance of both pain and suppuration. The teeth are firmer, but, owing to the great reduction in their means of attachment in consequence of the absorption of the sockets, they will continue loose.

"Another patient was cured under the same conditions. All the others happened to be in a much less advanced state of the disease.

"From the first, without exception, the pain ceased and the teeth have become firm, but the suppuration reappears from time to time, in some cases at very remote intervals. I think this fact may be attributed to two causes: first, that I did not leave the setons in place sufficiently long. Fearing some trouble, I thought it prudent to remove them from the fifth to the eighth day; but it seems to me there is no objection to keeping them in for several weeks. The second cause is the difficulty of the manual work. It is impossible to pass needles through the walls of the alveolus, and consequently to reach the lowest limit of the burrow. For this there must be invented a special instrument which I have not yet been able to make satisfactorily.

"A general treatment should be prescribed according to the case. I have obtained good effects from chloride of magnesium, whose action on the smooth fibre is most decided, and which produces besides a laxative effect almost always necessary. I propose to try, with the same object, injections of ergotine."

This ends my quotation, but I would say in closing that I have been asked several times whether, after three years' experience, I have still the same confidence in constitutional treatment in connection with local treatment that I had in the beginning. I can say most emphatically that the success with which many patients have been rewarded has been most satisfactory. I am firmly convinced that in a very large proportion of cases of pyorrhœa, where they have not gone on to the extent of the teeth loosening all hold, by the sockets being completely destroyed, they are benefited by careful constitutional treatment, embracing proper diet and the application of proper remedies in connection with local treatment. So that every day I am applying these remedies, and the patients say they are compensated fully in doing so.

